# Thermal, structural and acetylation behavior of snail and periwinkle shells chitin

**DOI:** 10.1007/s40204-017-0070-1

**Published:** 2017-07-19

**Authors:** Oluwashina Philips Gbenebor, Emmanuel Isaac Akpan, Samson Oluropo Adeosun

**Affiliations:** 1 Department of Metallurgical and Materials Engineering, University of Lagos, Lagos, Nigeria; 2 Institut für Verbundwerkstoffe GmbH, 67663 Kaiserslautern, Germany

**Keywords:** Chitin, Chitosan, Macromolecules, Deacetylation, Biofillers

## Abstract

This article reports a successful removal of CaCO_3_ from snail and periwinkle shells for the purpose of producing high quality chitin for possible application as bio-fillers in bone fixation materials. Experiment was designed with varying concentrations of acid and alkali for demineralization, deproteinization and deacetylation of the samples. Thermal characteristics, morphology, degree of de-acetylation, crystalline structure and hydrogen bonding characteristics of the extracted chitin were examined. Infra-red spectra, thermogravimetric analysis and X-ray diffraction patterns show that demineralization with 1.7 M HCl led to a successful removal of CaCO_3_. Subsequent deproteinization and deacetylation with 1.2 M NaOH led to a development of chitosan having a degree of deacetylation of 77 and 60% for periwinkle and snail shells, respectively. Generally, all results show that different treatments led to different chitin structure and consequently different properties.

**Electronic supplementary material:**

The online version of this article (doi:10.1007/s40204-017-0070-1) contains supplementary material, which is available to authorized users.

## Introduction

Waste materials from food processing which occur in large quantities can lead to environmental and human health problems (Hamed et al. 2016). However, these wastes can be turned into high value useful products. Periwinkle and snail shells wastes like other Crustaceans and Molluscs contain some amounts of chitin (Maruthiah and Palavesam 2017; Rasti et al. 2017; Ramasamy et al. 2017; Hamdi et al. 2017; Ehrlich et al. 2017). This chitin can be extracted and used for applications such as cosmetics, preservatives (Farajzadeh et al. 2016), surgical sutures (Usman et al. 2016), bio-composites (Petrenko et al. 2017), papermaking, water purification (Al-Manhel et al. 2016), photography (Muzzarelli 1997), metal uptake from wastewater (Nair and Madhavan 1984; Peniche-Covas et al. 1992) and solid-state batteries (Arof et al. 1995).

Snail and periwinkle shells are abundant in Nigeria. Successful extraction of chitin from these sources would convert seemly waste shells to wealth. The chitin so extracted can find usefulness as fillers in bio-polymer composite materials. These biodegradable and biocompatible composites can be used as temporal fixation materials for bone implants. However, these shells have been reported (Isa et al. 2012) to contain very high amount of CaCO_3_ and this makes it difficult to obtain high purity chitin derivative from them.

This research was designed based on the following objectives: (1) to investigate the possibility of removing CaCO_3_ from snail and periwinkle shells of Nigerian origin, so as to successfully extract chitin (2) to study the progress of deacetylation of chitin from these sources by monitoring structural changes as the concentration of the reagents are changed, (c) to establish a synergy between acid and alkali concentrations that will lead to a combination of high DD, high crystallinity and good thermal stability of the chitin. A three level full factorial experimental design was used for the study.

## Materials and methods

### Chitin extraction

Shells of periwinkle [*Tympanotonus fusatus* (L.)] and snail [*Achatina fulica* (L.)] were scraped free of loose tissues, washed in water, dried and ground to *2*50 μm in a ball mill. Demineralization was carried out at room temperature (32 °C) by soaking 100 g of ground samples in 1.5, 1.7 and 1.9 M HCl. For each concentration, the process was repeated several times until evolution of gaseous effluent ceased. The demineralized samples were washed with distilled water to neutral (pH 7.0), filtered and dried in the oven at 70 °C for 4 h to constant weights. Deproteinization was conducted by heating demineralised samples in 0.4, 0.8 and 1.2 M NaOH solutions in a beaker at 100 °C for 1 h. Deproteinized samples were filtered and soaked in fresh sets of alkali solutions (0.4, 0.8 and 1.2 M) for 18 h at room temperature (32 °C) for effective protein removal. These samples were then washed in distilled water to neutrality and oven dried at 70 °C. Depigmentation and bleaching was carried out by soaking the dried samples in 1 M H_2_O_2_ for 24 h at 32 °C. The resulting samples were washed in distilled water and dried for 4 h in an oven at 70 °C.

### X-ray diffraction (XRD)

The XRD of the samples was conducted using PAN analytical X’ Pert PRO MPD X-ray diffraction system PW3040/60 machine at Soochow University, Suzhou, China. Samples were exposed to a monochromatic Cu Kα radiation (*λ* = 1.5406 Å), operating at 40 kV and 40 mA. The samples were registered in a zero background sample holder to avoid external background interferences. The diffractograms were registered in the range of 7°–90° (2*θ*) in a step scan mode of 0.026261 at a counting time of 17.34 s per step.

### Fourier transform infrared spectroscopy (FTIR)

A Nicolet 6700 M spectrometer from Redeemers University, Ede, Nigeria, was used in carrying out FTIR spectra of samples in transmission mode. Ten milligram of fine samples were dispersed in a matrix of KBr (500 mg), followed by compression at 22–30 MPa to form pellets. The transmittance measurements were carried out in the range 400–4000 cm^−1^ at a resolution of 4 cm^−1^.

#### Hydrogen bond

Broad and overlapped FTIR absorption spectra existing between 3600 and 3000 cm^−1^ were resolved and improved by their deconvolution from a background scattering using a Gaussian function curve-fitting analysis with an *R* ^2^ > 0.99. The energy of the hydrogen bond E_H_ (kcal) was calculated using Eq. 1 as adopted by Gbenebor et al. (2016).

1 $$ E_{\text{H}} = \frac{1}{k}\left[ {\frac{{v_{\text{o}} - v}}{{v_{\text{o}} }}} \right], $$

where $v_{\text{o}}$ is the standard frequency corresponding to free OH groups (3600 cm^−1^); $v$ is the frequency of the bonded OH groups and *k* = 1.68 × 10^−2^ kcal^−1^.

#### Degree of deacetylation (DD)

The degree of deacetylation (DD) of the chitin samples was calculated using the baselines proposed by Domszy and Roberts (1985) given in Eq. 2.

2 $$ {\text{DD}} = 100 - \left[ {\frac{{(A_{1655} /A_{3450} ) \times 100}}{1.33}} \right] $$

The A_1655_ and A_3450_ were the absorbance at 1655 cm^−1^ of the amide I band as a measure of the N-acetyl group content and 3450 cm^−1^ of the hydroxyl band as an internal standard. The factor ‘1.33’ is the ratio of A_1655_/A_3450_ for fully N acetylated chitin.

### Thermogravimetric analysis (TGA)

In TGA, the change in mass of a sample is usually measured as a function of temperature or time under an inert (nitrogen) or oxidative (air) atmosphere. TGA was used to quantitatively determine the constituent of minerals and organic compound (including the biopolymer) in the samples. Analysis of samples was carried out on TGA Q500 instrument in Soochow University, Suzhou, China. 2 mg of samples was heated to 750 °C at 10°/min heating rate. In this test, the temperature for the onset of thermal decomposition (*T*_onset_), the temperature at which decomposition rate was rapid (*T*_max_), chitin content and yield were deduced from the thermograms.

### Scanning electron microscopy (SEM) with energy dispersive X-ray analysis (EDS)

An ASPEX 3020 model variable pressure SEM from Soochow University, Suzhou, China operated with an electron intensity beam 15 kV and equipped with Noran-Voyager energy dispersive spectroscope (EDS) was used to observe the morphological features of all samples. The samples to be observed under the SEM were mounted on a conductive carbon imprint left by the adhesive tape prepared by placing the samples on the circular holder and coated for 5 min to enable it conduct electricity.

## Results and discussion

### De-acetylation progress of periwinkle shell chitin

**Fig. 1:**
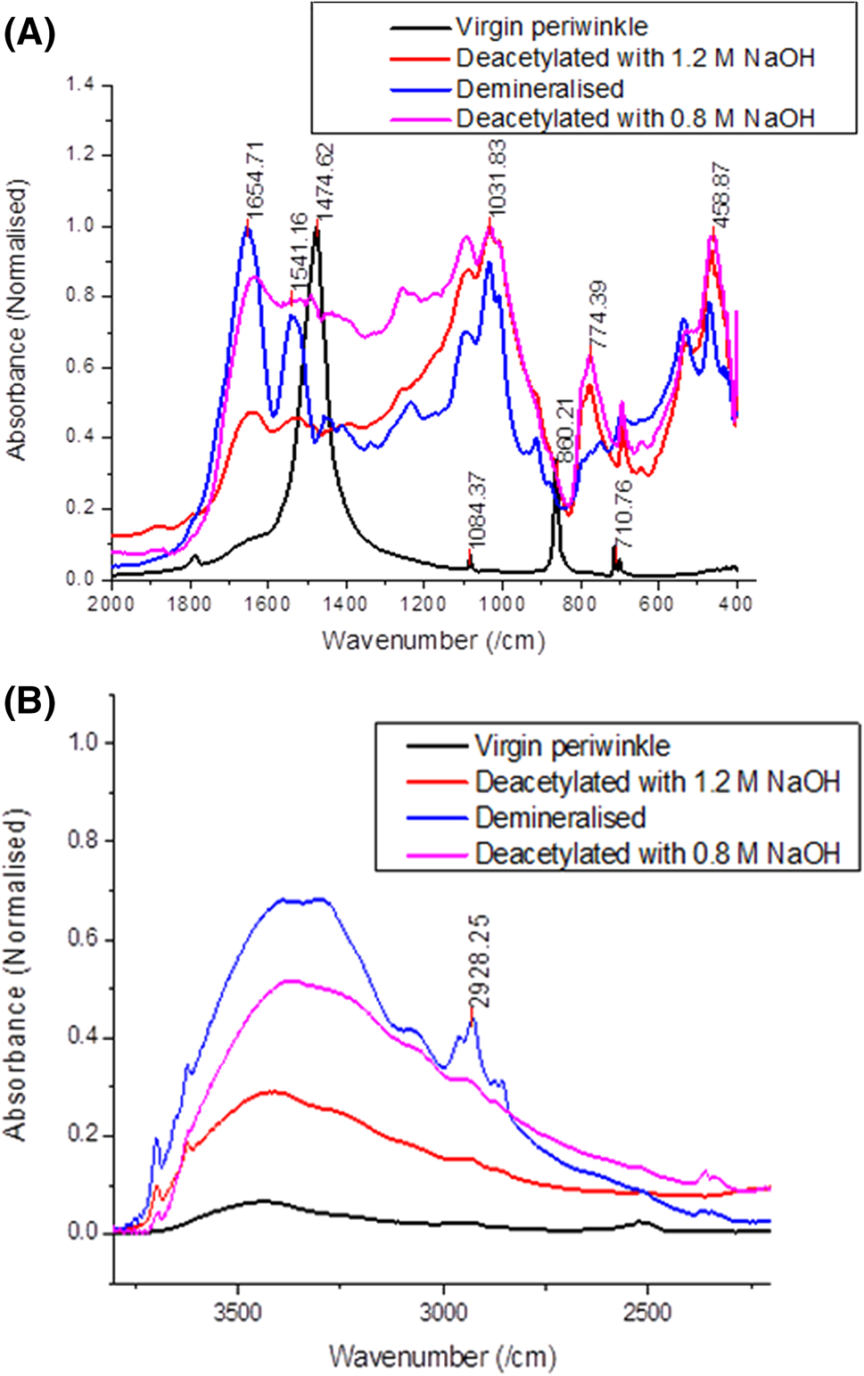
De-acetylation progress of periwinkle shell chitin

The process of de-acetylation involves the removal of acetyl groups from the molecular chain of chitin, leaving behind the amino group (–NH_2_). Properties such as solubility, viscosity, ion-exchange capacity, flocculation ability, tensile strength, ability to chelate metal ions, immune-adjuvant activity and reaction with amino group are all dependent on DD (Zhang et al. 2011; Yuan et al. 2011; Kasaai 2009; Tan et al. 1998). FTIR was used to study the removal of acetyl groups and the development of amino group during de-acetylation of the samples. Figures 1a and b show comparative FTIR spectra of samples with different treatment conditions. The figure reveals changes in the functional group with respect to treatment. The untreated sample shows the presence of bands at 1474 and 860 cm^−1^ indicating the presence of calcite (Gbenebor et al. 2016; Udomkan and Limsuwan 2008; Rahman and Halfar 2014). Other low intensity peaks also appear which have been associated with the presence of ${\text{CO}}_{3}^{ - 2}$(Aku et al. 2012). These peaks disappeared when the shells were acid treated, giving rise to a chitin structure (see Fig. 1a) with strong amide I and II peaks at 1654 and 1541 cm^−1^ (Kaya and Baran 2015).

The band between 2500 and 3600 cm^−1^ also greatly increased (see Fig. 1b). This increase is attributed to the increase in hydrogen bond energy. α-chitin is known to possess strong inter-sheet and intra-sheet hydrogen bonding which is responsible for its stability and poor solubility (Rudall and Kenchington 1973; Khan et al. 2002). Other bands indicating the development of saccharide rings and N–H stretching also appear after the acid treatment.

The spectrum of deproteinized samples reveals a marked reduction in the C=O secondary amide stretch at 1654 cm^−1^ (amide I). However, the N–H bend and C–N stretch at 1541 cm^−1^ (amide II) increased. This is an indication that de-acetylation occurred leading to increase in amount of NH_2_ (This suggests an increase in DD). This scenario is supported by the decrease in the band between 2500 and 3600 cm^−1^ in Fig. 1b. It is widely believed that increase in DD is always accompanied by a decrease in inter-sheet and intra-sheet hydrogen bond energy.

It is also evident from Fig. 1a that increase in concentration of NaOH led to further decrease in C=O secondary amide stretch at 1654 cm^−1^ (amide I). The N–H bend, NH out of plane bending at 774 cm^−1^ and C–N stretch at 1541 cm^−1^ (amide II) increases, indicating an increase in the amount of NH_2_. Figure 1b shows a decrease in OH band height showing further decrease in hydrogen bonding. Table 1 shows the effect of change in concentration of alkali and acid on the values of DD. It is evident that increasing alkali concentrations led to increase in DD. This was not the case when acid concentration was increased.

**Table 1:** Degree of de-acetylation of extracted chitin

Treatment conditions	DD (%)
1.5 M HCl + 0.4 M NaOH	52
1.5 M HCl + 0.8 M NaOH	61
1.5 M HCl + 1.2 M NaOH	67
1.7 M HCl + 0.4 M NaOH	53
1.7 M HCl + 0.8 M NaOH	64
1.7 M HCl + 1.2 M NaOH	77
1.9 M HCl + 0.4 M NaOH	64
1.9 M HCl + 0.8 M NaOH	55
1.9 M HCl + 1.2 M NaOH	63

### OH bonding for periwinkle shell chitin

**Fig. 2:**
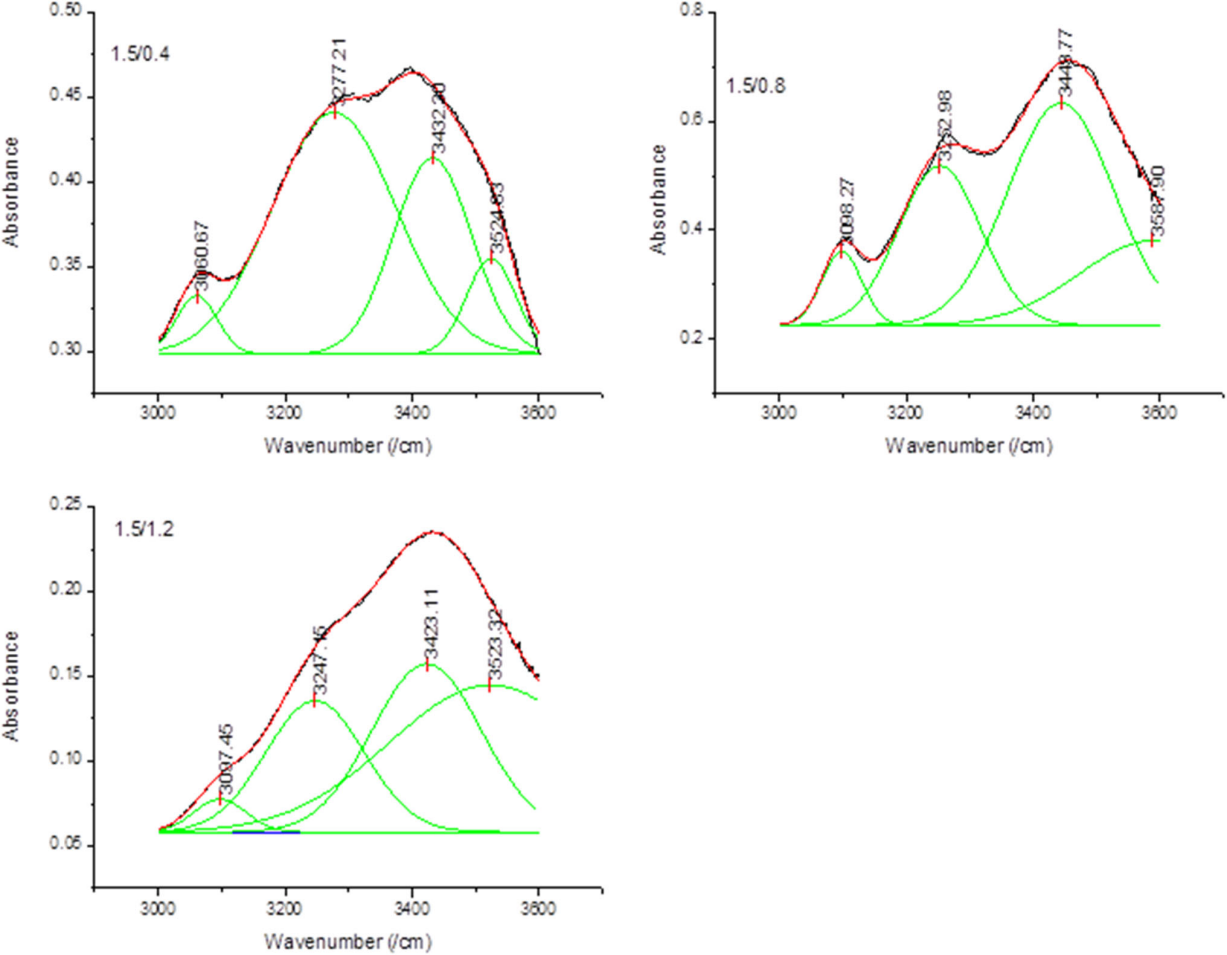
OH bonding for treated periwinkle chitin with 1.5 M HCl (figures for 1.7 and 1.9 M HCl treated samples are given in supplementary data file)

A careful analysis of the FTIR spectra (Fig. 1) show that all treated samples exhibited a splitting of the primary amide I band. This is a sufficient proof that the chitins obtained are α- structure (Sikorski et al. 2009; Wang et al. 2013). It is widely acknowledged that, α-chitin is stabilized by two intra-molecular hydrogen bonds (C(3)–OH···O–C(5) and C(6)–OH···OC), and two intermolecular hydrogen bonds, NH···OC and C(6)–OH···OH–C(6) (Kameda et al. 2005; Minke and Blackwell 1978). In this study, the original FTIR spectra were broad and difficult to distinguish separately. Mathematical best fits were applied to the curves after normalization using the Gaussian function (Fig. 2). The fitting was performed until *R* ^2^ > 0.998 was obtained. Band assignment was performed according to the information obtained from Liu et al. (2010), Hu et al. (2007), Sikorski et al. (2009) and Kameda et al. (2005). The average content (% proportion) of each band in the OH-band network was calculated in accordance to Gbenebor et al. (2016). Generally, it was observed that NH–OC and C(3)OH–O–C bands were the dominant bands in the hydrogen bonding network. For 1.5 M HCl/0.4 M NaOH (group I) treated samples, NH—OC band occupied the largest proportion (57%) of the entire OH-band. In the case of 1.5 M HCl/0.8 M NaOH (group II) treated samples C(3)–OH–O–C occupied the largest proportion (49%) of the entire OH-band. Similar to group I, NH–OC occupied the largest proportion (33%) in 1.5 M HCl/1.2 NaOH (group III) samples. A similar trend was noticed in 1.7 M HCl and 1.9 M HCl treated samples. A higher amount of these bands is an indication of higher stability. Table 2 shows that, the energy of the hydrogen bond (*E*_H_) was higher for intermolecular bonds compared to intra-molecular bonds.

**Table 2:** Inter-sheet hydrogen bond energy for periwinkle shell chitin

	Sample treatment	C(6)–OH···OH–C(6)	C(3)–OH···O–C(5)	Average bond energy
		Bond energy (kcal)	Bond energy (kcal)	Bond energy (kcal)
1.	1.5 M HCl + 0.4 M NaOH	8.92	2.77	11.69
2.	1.5 M HCl + 0.8 M NaOH	8.30	2.58	10.88
3.	1.5 M HCl + 1.2 M NaOH	8.31	2.92	11.23
4.	1.7 M HCl + 0.4 M NaOH	9.18	2.81	11.99
5.	1.7 M HCl + 0.8 M NaOH	8.26	3.24	11.50
6.	1.7 M HCl + 1.2 M NaOH	8.14	2.95	11.09
7.	1.9 M HCl + 0.4 M NaOH	8.85	3.25	12.10
8.	1.9 M HCl + 0.8 M NaOH	9.26	3.02	12.28
9.	1.9 M HCl + 1.2 M NaOH	8.57	2.99	11.56

It is widely acknowledged that inter-sheet and intra-sheet hydrogen bonding is responsible for basic chitin properties such as solubility, crystallinity and strength (Kurita et al. 1994; Kurita et al. 1990; Kurita et al. 1993b; Kurita et al. 1993a; Rudall and Kenchington 1973). However, out of the four hydrogen bonds (Liu et al. 2010; Hu et al. 2007; Sikorski et al. 2009; Kameda et al. 2005) identified in chitin, C(3)–OH···O–C(5) and C(6)–OH···OC are known to be responsible for the stability, solubility and crystallinity (Gbenebor et al. 2016). The average bond energy of these bonds is a measure of the intra-sheet bond strength. This bond energy can be used as a relative measure of the effect of treatment on chitin structure. Therefore, the best combination of treatment to achieve desired properties can be determined by (1) understanding the development of this bond energy, and (2) correlating the bond strength with DD. Table 2 shows the average intra-sheet bond energy. The average bond energy was found to decrease with increase in alkali concentration. It is also important to note that higher concentration of acid leads to higher hydrogen bond energy. On the other hand, samples with higher DD exhibits relatively lower average bond energy.

### Crystalline structure of periwinkle chitin

**Fig. 3:**
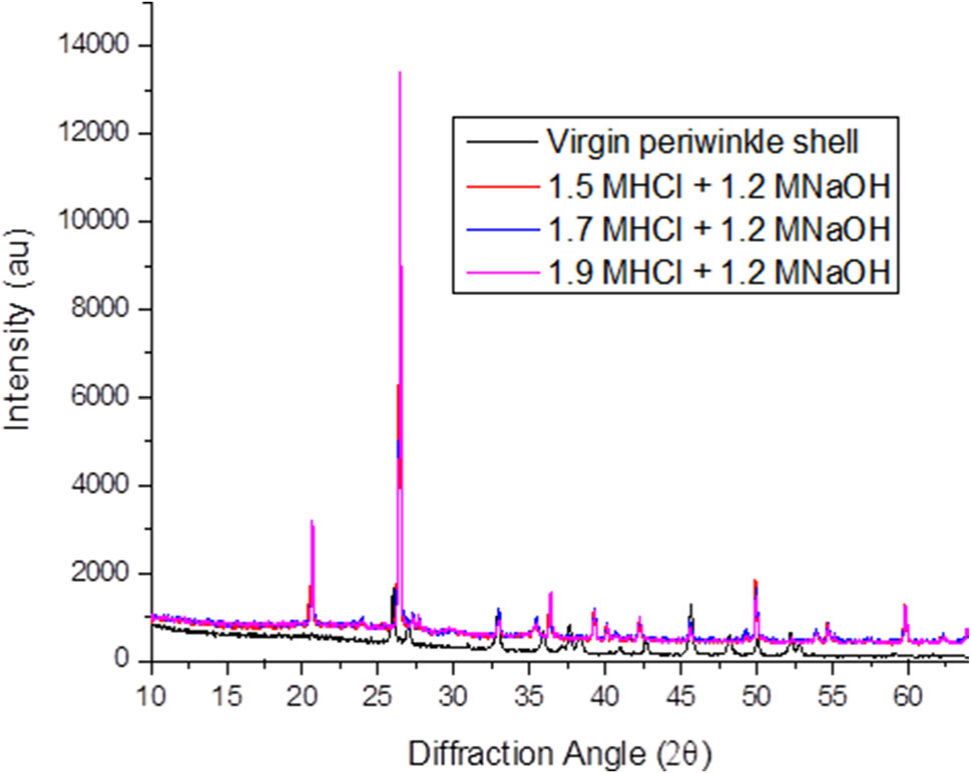
XRD of virgin periwinkle and selected chitin samples

Figure 3 shows the X-ray diffraction patterns of untreated periwinkle shell and extracted chitins. The figure shows that prominent chitin peaks were developed after treatments. The diffraction peaks at 27.07, 35.84 and 42.72 in the virgin sample are crystalline diffractions from calcite (Mikkelsen et al. 1997; Heredia et al. 2007). These peaks are not present in the treated samples, showing that the treatment was successful in removing CaCO_3_ from the shells. The XRD patterns for treated samples show two sharp crystalline reflections at 20.6° and 26.4° which are typical reflections of de-acetylated chitin (Cho et al. 2000; Jang et al. 2004; Yen et al. 2009; Sajomsang and Gonil 2010; Gonil and Sajomsang 2012; La Juárez-de Rosa et al. 2012). Peaks occurring in the 20.5–20.8° range are attributed to (020) of chitosan and the peaks in the 26.3–26.6° diffraction range are attributed to (013) of α-chitin. This indicates that the treated samples contain a combination of chitin and chitosan. The figure also shows that different treatment led to different diffraction intensities. This is an indication of difference in structure of the samples due to difference in treatment.

### Thermal properties of periwinkle chitin

**Fig. 4:**
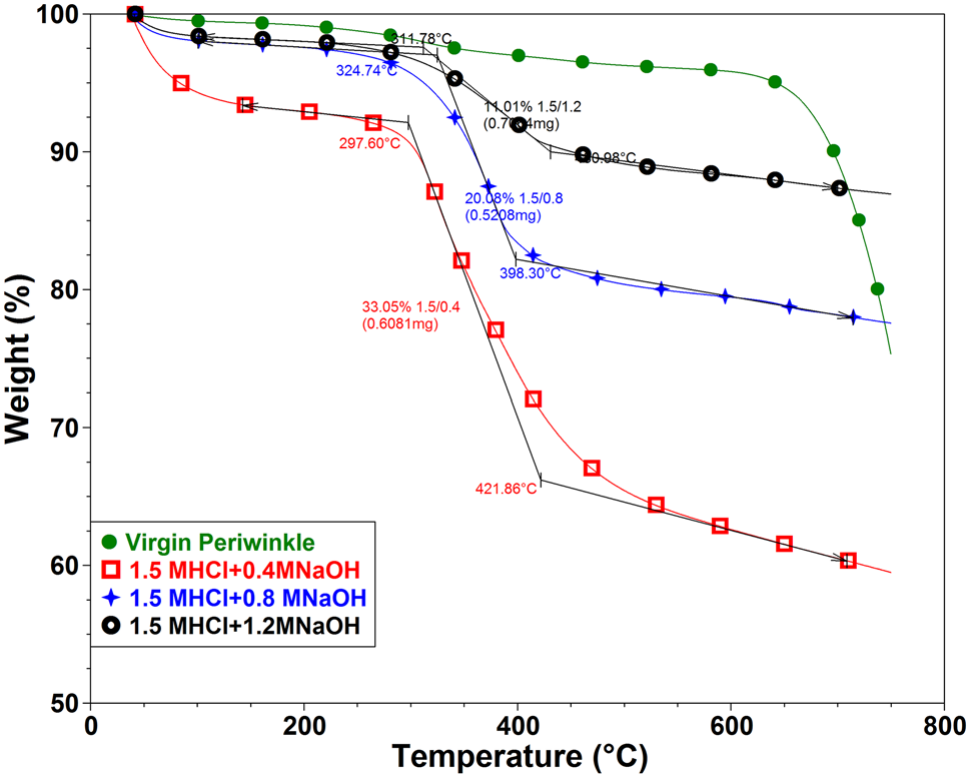
TGA of periwinkle chitin treated with 1.5 M HCl and varying alkali concentration (other TGA figures are shown in the supplementary data file)

**Fig. 5:**
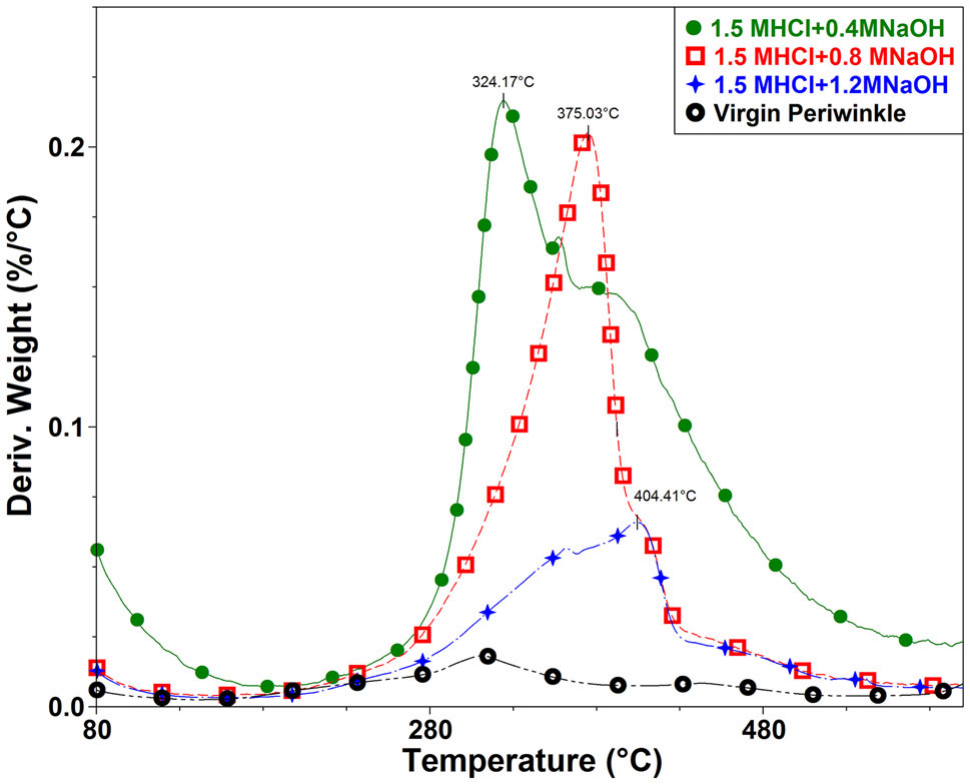
DTG of periwinkle chitin treated with 1.5 M HCl and varying alkali concentration

Thermal stability is a critical factor for the determination of potential applications of chitin and its derivatives. Chitin requires high thermal energy for dissociation of its crystalline structure. This makes thermal characterization an important aspect of chitin characterization (Wang et al. 2013). This section deals with the effect of treatment on the thermal behavior of chitin extracted from periwinkle shell. Figures 4 and 5 are representative TGA and DTG curves of treated and virgin samples. Figure 4 shows that the thermal degradation of 1.5 M HCl treated samples proceed in two stages. This is typical of chitin extracted from natural sources (Abdou et al. 2008; Jang et al. 2004; Paulino et al. 2006; Sagheer et al. 2009; Wanjun et al. 2005). The first weight loss which occurred between 30 and 100 °C is attributed to the loss of absorbed or bounded water (Zakaria et al. 2012). This accounted for 4.35, 0.48 and 0.73% weight loss in 0.4, 0.8 and 1.2 M NaOH-treated samples, respectively. These values are quite small compared to those obtained by (Kaya et al. 2013; Kaya et al. 2015a; Kaya et al. 2014; Zakaria et al. 2012; Shigemasa et al. 1996; Sajomsang and Gonil 2010; Sagheer et al. 2009; La Juárez-de Rosa et al. 2012).

The second stage of degradation occurs between 290 and 430 °C with 33, 20 and 11% weight loss as for 0.4, 0.8 and 1.2 M NaOH-treated samples, respectively. This decomposition is attributed to the decomposition of saccharide backbones, polymerization and decomposition of acetylated and deacetylated units of chitin (Sajomsang and Gonil 2010; Sagheer et al. 2009). The onsets of degradation for 0.4, 0.8 and 1.2 M NaOH-treated samples are 297, 324 and 311 °C, respectively. The DTG_max_ of 0.4, 0.8 and 1.2 M NaOH-treated samples are 324, 375 and 404 °C, respectively (Fig. 5). Jang et al. (2004) recorded similar values for alpha and beta chitin in their study. The difference in the peak temperature is attributed to the difference in intermolecular hydrogen bonds of the samples. This indicates a difference in structure as also seen in results of the FTIR presented in previous section. Thermal decomposition activation energies are 60.83, 50.13 and 54.05 kJ mol^−1^ for 0.4, 0.8 and 1.2 M NaOH-treated samples, respectively. Generally, the 0.8 M NaOH-treated samples shows the best thermal stability. A similar trend was noted in 1.7 and 1.9 M HCl treated samples. However, differences in activation energy, onset of degradation and peak degradation temperatures were noted (Table 3). These differences, proves the difference in structure of the treated samples which has been attributed to the difference in treatment conditions.

**Table 3:** Thermal properties of periwinkle chitin

Sample	Activation energy	Residue at 600 °C	DTG2 (%)	Water (%)	DTGmax	Onset temp
1.5 M HCl + 0.4 M NaOH	60.83	62.60	33.05	4.35	324.17	297.06
1.5 M HCl + 0.8 M NaOH	50.13	79.44	20.08	0.48	375.03	324.00
1.5 M HCl + 1.2 M NaOH	54.05	88.26	11.01	0.73	404.41	311.78
1.7 M HCl + 0.4 M NaOH	66.61	69.46	27.56	2.98	343.78	306.00
1.7 M HCl + 0.8 M NaOH	9.23	91.17	8.04	0.79	382.90	327.52
1.7 M HCl + 1.2 M NaOH	68.29	87.31	11.85	0.84	388.17	327.75
1.9 M HCl + 0.4 M NaOH	61.52	51.92	41.20	6.88	318.50	296.35
1.9 M HCl + 0.8 M NaOH	51.24	77.30	20.56	2.14	395.05	337.74
1.9 M HCl + 1.2 M NaOH	44.41	84.61	12.06	3.33	390.39	346.41

Generally, it is noted that samples treated with 0.4 M NaOH present a DTG with two or more peaks in the second degradation stage. This behavior was also noted by Georgieva et al. (2012). They suggested that the first peak is associated with thermal depolymerisation of chitin and the formation of volatile low molecular weight product whereas the second peak is attributed to oxidative thermal degradation of formed char. This occurrence confirms the difference in degradation behavior of chitin extracted with different treatment conditions. On the other hand, it is also noted that in all cases samples treated with 0.4 M NaOH possess the highest and broadest peak.

### Surface morphology of chitin extracted from periwinkle shell

**Fig. 6:**
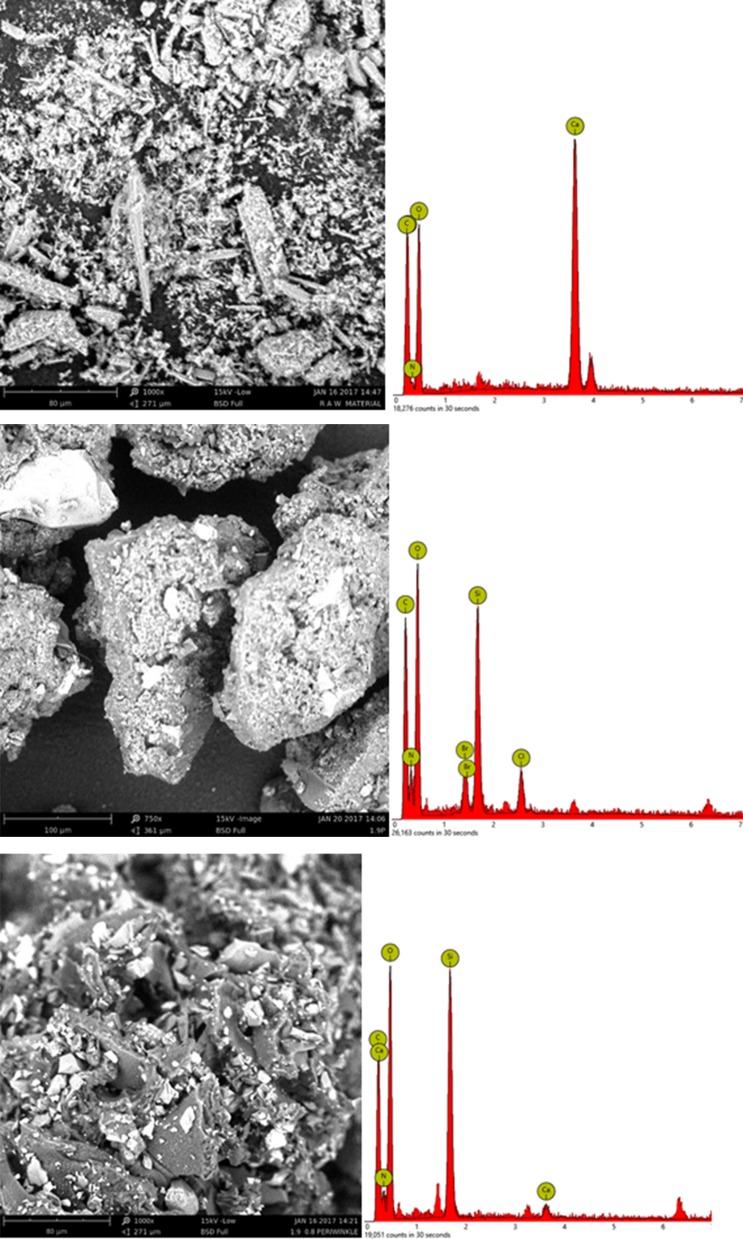
SEM/EDS images of periwinkle shell chitin **a** untreated, **b** acid treated **c** 1.9 M HCl/0.8 M NaOH treated

Changes in surface morphology of the chitin in relation to treatment conditions are shown in Fig. 6. Figure 6a is the SEM image of raw periwinkle shell. The image shows non-uniform sized particles with rough surfaces. The rough surface is an indication of the prevalence of CaCO_3_ in the parent material. As shown in the EDS, calcium-related compounds constitute the highest percentage in the tested sample. Demineralised sample (Fig. 6b) shows agglomeration with evidence of the development of fibrillar structure. The fibrillar structure is attributed to the high OH bonding in the extracted chitin. The EDS shows the absence of calcium in the demineralised sample. This shows that demineralisation led to removal of the CaCO_3_. The EDS also show increase in carbon and hydrogen content. This confirms the development of chitin structure. Figure 6c shows the effect deacetylation with 0.8 M NaOH. The images shows plate-like micro-fibrillar structure changes to globular structure. This indicates the action of deacetylation.

### De-acetylation progress of snail chitin

**Fig. 7:**
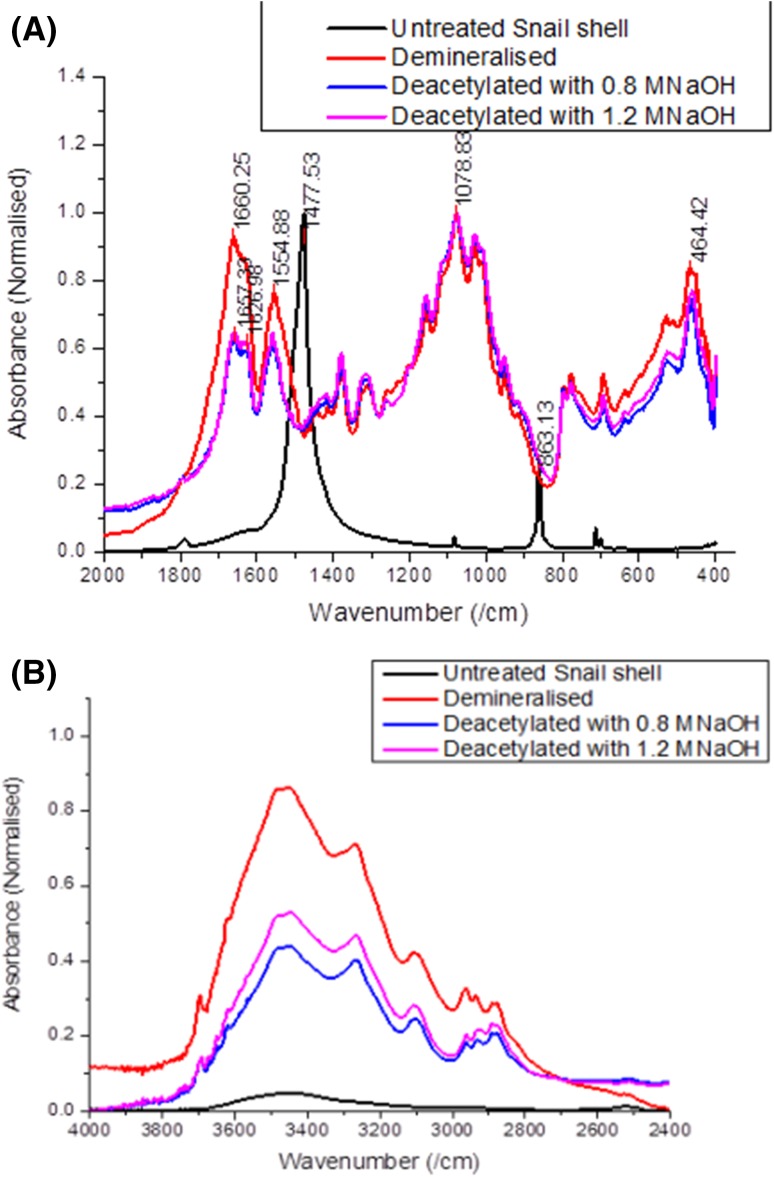
De-acetylation Progress of periwinkle shell chitin

Figures 7a and b show the difference in FTIR spectra of untreated and treated snail shells. The starting material (untreated sample) show prominent absorptions peaks at 1477 and 863 cm^−1^. These are attributed to CaCO_3_ adsorption (Gbenebor et al. 2016; Udomkan and Limsuwan 2008; Rahman and Halfar 2014). Other low intensity peaks also appear at 1786 and 714 cm^−1^,which are attributed to the presence of ${\text{CO}}_{3}^{ - 2}$(Udomkan and Limsuwan 2008). It is evident from the figure that these peaks are absent in the spectra of all treated samples. The absence of these peaks is an indication of the effectiveness of the demineralisation process to remove the unwanted CaCO_3_. The spectrum of demineralized sample show the presence of polysaccharide rings, secondary amides and NH links (1660, 1554, 1078, 464 cm^−1^, etc.). This indicates the development of chitin structure (Kaya and Baran 2015; Kaya et al. 2015b; Kaya et al. 2014). The N–H and O–H stretching (3100–3600 cm^−1^ range) experienced an increase in peak height after demineralization. Subsequent treatment with 0.8 M NaOH brought about a marked change in amide I, II and O–H, N–H bands.

The secondary amide I band at 1660 cm^−1^ and the amide II band at 1554 cm^−1^ experience significant decrease. The amide I band broke into two low frequency bands (1657 and 1626 cm^−1^). The splitting of this band indicates that the treated sample is an alpha structure chitin. The figure also shows that O–H, N–H bands (3100–3600 cm^−1^) narrows and decrease markedly after treatment with 0.8 M NaOH. This is an indication that deacetylation led to a decrease in inter/intra sheet H-Bond energy. Further treatment with 1.2 M NaOH did not produce so much change in the spectra except a marginal change in the height of O–H, N–H bands. This indicates that further de-acetylation led to a slight change in the structure, which may be attributed to depolymerisation of the polysaccharide.

### OH bonding for snail shell chitin

**Fig. 8:**
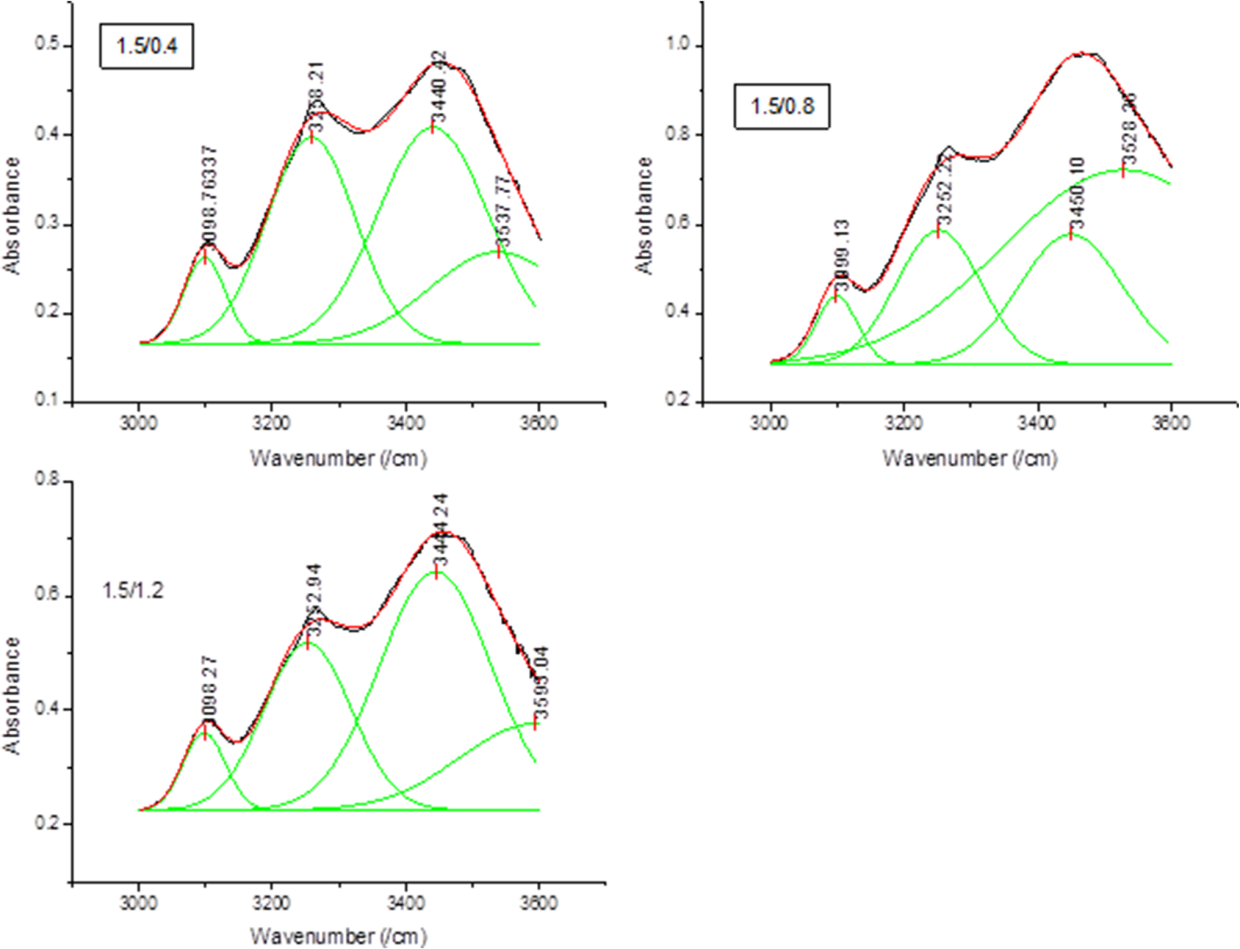
OH bonding for snail chitin treated with 1.5 M HCl and **a** 0.4, **b** 0.8 and **c** 1.2 M NaOH (figures for samples treated with 1.7 and 1.9 M HCl are given in the supplementary data file)

The results of spectral fitting of the OH broad band of snail shell chitin samples are shown in Fig. 8. Samples treated with 1.5 M HCl/0.4 M NaOH show inter and intra-molecular bonds of C(6)–OH···OH–C(6), NH···OC, C(3)–OH···O–C(5) and C(6)–OH···OC at 3098, 3258, 3440 and 3537 cm^−1^, respectively. For this sample, C(3)–OH···O–C(5) is found to occupy the highest proportion (42.68%) of the entire OH bond. The higher proportion of the C(3)–OH···O–C(5) intra chain bonding is an indication of superior chain stability (Kumirska et al. 2010). An increase in alkali concentration to 0.8 M NaOH brought a decrease in the proportion of C(3)–OH···O–C(5) to 22.59%, but significant increase in the C(6)–OH···OC (52.92%). However, the proportions of NH···OC and C(6)–OH···OH–C(6) inter chains decreased. This decrease points to increase in DD (Cho et al. 2000). A further increase in alkali concentration to 1.2 M NaOH gave a profound increase in C(3)–OH···O–C(5) intra chain bonds (51.15%).

Increase in acid concentration to 1.7 M presents a slightly different result. The C(3)–OH···O–C(5) inter-chain is found to dominate the OH bonding in all cases. Treatment with 0.4, 0.8 and 1.2 M NaOH resulted in 61, 58, and 66% of C(3)–OH···O–C(5), respectively. However, the relative proportion of C(6)–OH···OH–C(6) and NH···OC was lower in all cases. The low amount of these bonds implies that samples undergo transformation from anti-parallel to parallel sheet arrangement, which point to increase in DD. This is evident in Table 4 which reveals that the DD of these samples are slightly >50% (Kumirska et al. 2010).

**Table 4:** Degree of deacetylation of chitin extracted from snail shell

Sample treatment	DD (%)
1.5 M HCl + 0.4 M NaOH	53
1.5 M HCl + 0.8 M NaOH	51
1.5 M HCl + 1.2 M NaOH	60
1.7 M HCl + 0.4 M NaOH	52
1.7 M HCl + 0.8 M NaOH	58
1.7 M HCl + 1.2 M NaOH	56
1.9 M HCl + 0.4 M NaOH	50
1.9 M HCl + 0.8 M NaOH	46
1.9 M HCl + 1.2 M NaOH	56

Spectral fittings for samples treated with higher acid concentration (1.9 M) indicates that the treatment produced slight decrease in the amount of C(3)–OH···O–C(5) in all cases and a slight increase in amount of NH···OC and C(6)–OH···OH–C(6) in all cases. Thus, further increase in acid concentration does not produce significant changes. Table 5 shows the inter-sheet hydrogen bonding energy for snail shell chitin. The average hydrogen bond energy did not differ significantly from each other. However, comparing Tables 4 and 5, it is deduced that treatment combination that produces higher DD also leads to relatively lower average bond energy.

**Table 5:** Inter-sheet hydrogen bond energy for periwinkle shell chitin

S/N	Sample	C(6)–OH···OH–C(6)	C(3)–OH···O–C(5)	Average bond energy (kcal)
		Bond energy (kcal)	Bond energy (kcal)	
1.	1.5 M HCl + 0.4 M NaOH	8.29	2.64	10.93
2.	1.5 M HCl + 0.8 M NaOH	8.28	2.48	10.76
3.	1.5 M HCl + 1.2 M NaOH	8.30	2.58	10.88
4.	1.7 M HCl + 0.4 M NaOH	8.29	2.33	10.62
5.	1.7 M HCl + 0.8 M NaOH	8.26	2.45	10.71
6.	1.7 M HCl + 1.2 M NaOH	8.26	2.31	10.57
7.	1.9 M HCl + 0.4 M NaOH	8.28	2.38	10.66
8.	1.9 M HCl + 0.8 M NaOH	8.26	2.34	10.60
9.	1.9 M HCl + 1.2 M NaOH	8.26	2.36	10.62

### Crystalline structure of snail chitin

**Fig. 9:**
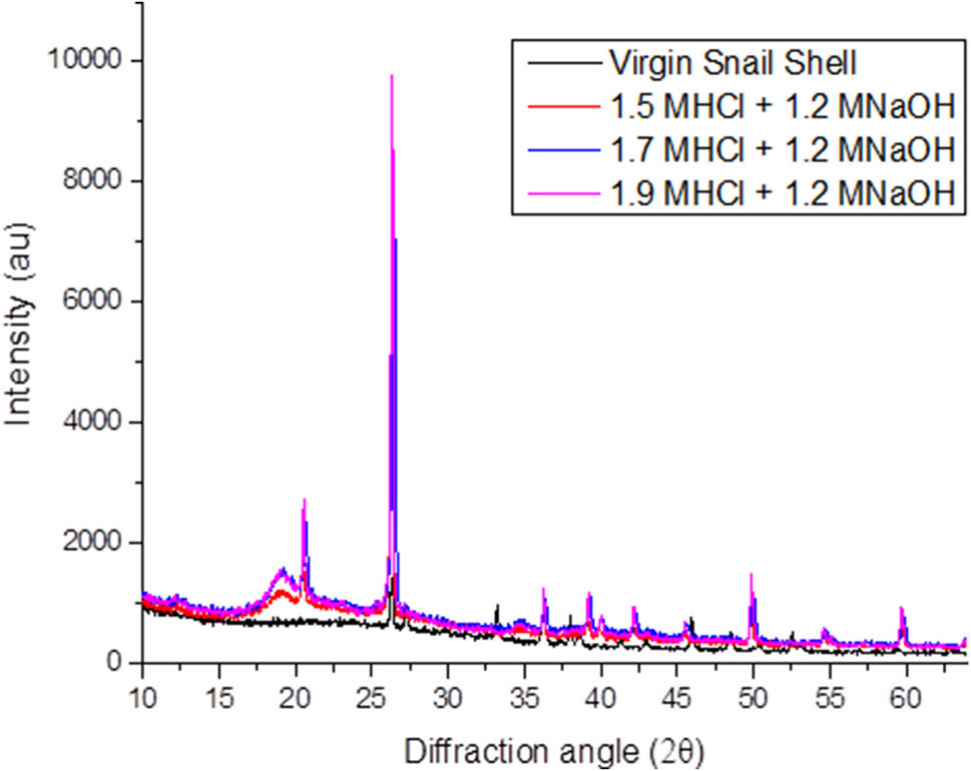
XRD of chitin from snail shell

The XRD patterns for virgin snail shell and extracted chitin samples are shown in Fig. 9. CaCO_3_ crystalline diffraction peaks are shown in the virgin samples at 27.32, 33.23, 36.29 and 43.07 (Heredia et al. 2007; Mikkelsen et al. 1997). These peaks are absent in the extracted chitin showing that the treatment was successful in removing CaCO_3_ from the shells. The XRD patterns of treated samples show prominent peaks between 20° and 21° and 26°–27°. A broad peak is also shown between 18° and 19° for the treated samples. A similar XRD pattern has been reported for crab, shrimp, krill and insects in earlier studies (Yen et al. 2009; Sajomsang and Gonil 2010; Wang et al. 2013; Liu et al. 2010; Liu et al. 2012). The broad peak between 18 and 19° is an evidence of de-acetylation (Jang et al. 2004; Sagheer et al. 2009) and confirms that the treated samples are deacetylated chitin. The peaks between 20.5° and 20.8° are attributed to (020) of chitosan whereas the peaks between 26.3°–26.6° and 18°–19° are attributed to (013) and (110) of α-chitin, respectively.

### Thermal properties of snail chitin

**Fig. 10:**
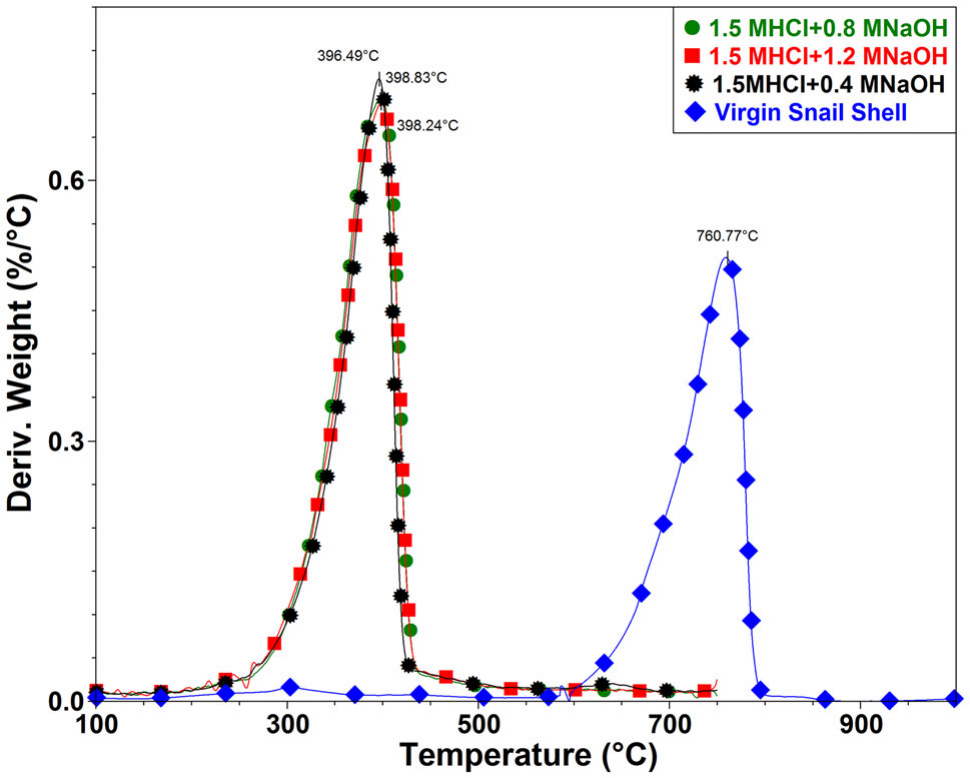
DTG of snail chitin treated with 1.5 M HCl and varying alkali concentration (DTG for 1.7 and 1.9 M HCl treated samples are given in supplementary data file)

**Fig. 11:**
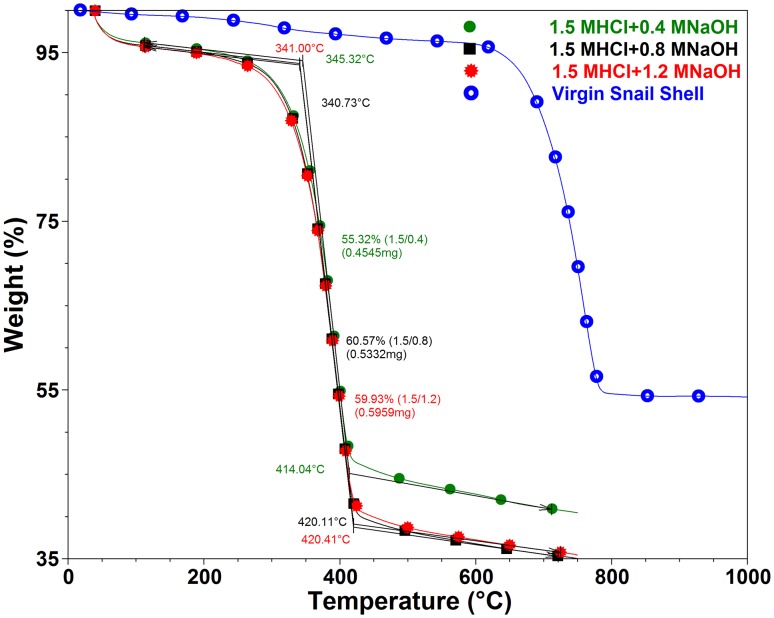
TGA of snail chitin treated with 1.5 M HCl and varying alkali concentration (TGA for 1.7 and 1.9 M HCl treated samples are given in supplementary data file)

Figures 10 and 11 show DTG and TGA curves for samples treated with 1.5 M HCl. Samples were first subjected to degradation test between 25 and 1000 °C to check for the degradation of CaCO_3_ which usually occur between 600 and 800 °C. As shown in Fig. 10 virgin snail shells show the degradation (42.38%) of CaCO_3_ between 600 and 800 °C. All treated samples did not show any degradation between 600 and 800 °C indicating that CaCO_3_ was completely removed during the treatment. After some confirmatory runs, it was decided that all treated samples be subjected to degradation between 25 and 750 °C temperature range. TGA curves of treated samples (Fig. 10) reveal that degradation occurs in two steps. This is in line with other studies reporting the extraction of chitin from other sources (Sagheer et al. 2009; La Juárez-de Rosa et al. 2012; Abdou et al. 2008). The first of these mass losses is attributed to evaporation of the water, and the second, to decomposition of the chitin structure (Wang et al. 2013; Paulino et al. 2006; Jang et al. 2004). The water content varied between 3.96 and 4.19% of the total mass depending on the treatment condition (Table 6). The mass loss is seen to increase with increase in alkali concentration.

**Table 6:** Thermal properties of snail shell chitin

Sample	Activation energy	Residue at 600 °C	DTG2	DTG1	DTGmax	Onset Temp
1.5 M HCl + 0.4 M NaOH	24.31	40.72	55.32	3.96	396.32	345.32
1.5 M HCl + 0.8 M NaOH	27.20	35.24	60.57	4.19	397.91	340.73
1.5 M HCl + 1.2 M NaOH	29.08	35.73	59.93	4.34	398.90	341.00
1.7 M HCl + 0.4 M NaOH	93.92	34.19	56.85	8.96	360.81	319.33
1.7 M HCl + 0.8 M NaOH	56.62	33.23	55.50	11.27	371.18	332.46
1.7 M HCl + 1.2 M NaOH	34.93	30.51	59.80	9.69	380.86	335.45
1.9 M HCl + 0.4 M NaOH	28.03	36.32	57.71	5.97	384.12	338.98
1.9 M HCl + 0.8 M NaOH	25.66	47.27	48.13	4.60	393.40	341.28
1.9 M HCl + 1.2 M NaOH	27.66	42.57	51.97	5.46	397.43	344.28

In the second step, the observed mass loss varied between 55 and 60% depending on treatment conditions (Table 6). The maximum mass loss (60.57%) is observed in the 0.8 M NaOH-treated sample whereas the minimum mass loss (55.32%) is observed in 0.4 M NaOH-treated sample. The DTGmax values (396–398 °C), are similar for all treated samples (Table 6). Other authors, reported DTGmax value of alpha chitin to vary between 350 and 400 °C (Wang et al. 2013; Sajomsang and Gonil 2010; Kaya et al. 2014). The calculated activation energies for the treated samples are 24, 27 and 29 kJ mol^−1^ for 0.4, 0.8 and 1.2 M NaOH-treated samples, respectively. This shows that increase in alkali concentration led to increase in thermal stability. The onsets of degradation for the samples are 345, 340 and 341 °C for 0.4, 0.8 and 1.2 M NaOH, respectively. These values are higher than those reported by Abdou et al. (2008), Gbenebor et al. (2016), Sagheer et al. (2009), Zawadzki and Kaczmarek (2010) and Shigemasa et al. (1996). The residue after 1000 ◦C is between 35 and 41% similar to that reported by Gbenebor et al. (2016) for crab and shrimp shell chitins. Table 6 summarizes the thermal properties for all the treated samples. A similar trend is noticed for each group with very minimal difference.

### Surface morphology of chitin extracted from snail shell

**Fig. 12:**
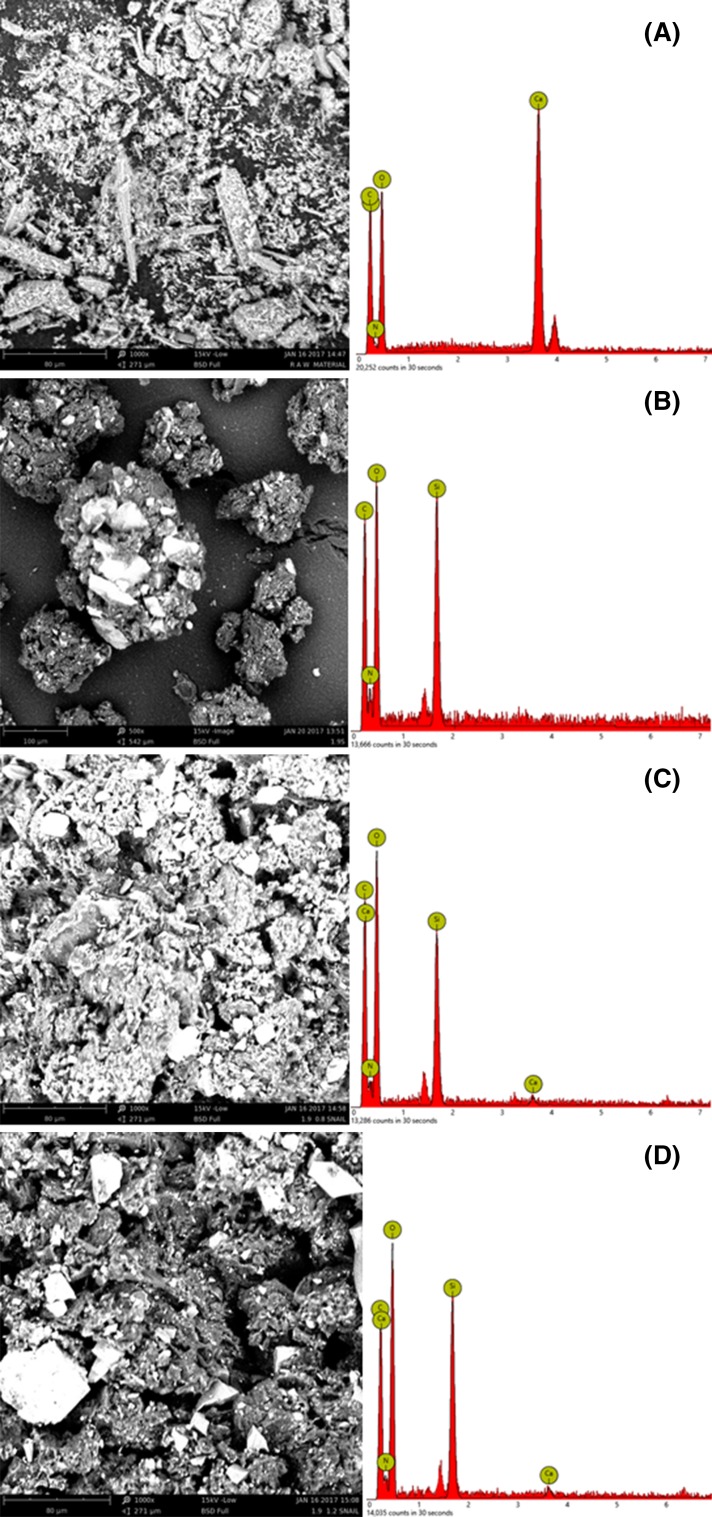
SEM images of snail shell chitin **a** untreated **b** Acid treated **c** 1.9 M HCl/0.8 M NaOH treated **d** 1.9 M HCl/0.8 M NaOH treated

Figure 12a is the SEM image of the virgin snail shell showing non-uniform particle size with rough surface. The EDS shows that the sample contains very high percentage of calcium. Treatment with 1.9 M HCl (see Fig. 12b) led to a change from rough to smooth surface. This is attributed to the removal of CaCO_3_. The EDS of this sample shows the absence of calcium. This indicates that calcium-related compounds were completely removed after demineralisation. Carbon and nitrogen increased indicating that brought about the development of chitin structure. On the other hand, the EDS shows that Si is present in the demineralised sample. This was not seen in the virgin sample. The presence of Si in crustacean shells has been reported by Isa et al. (2012). The globular white-like particles are identified as oxygen containing mineral compounds. EDS show that Si and oxygen are the major component of this particle. The dark-like particles are identified as carbon containing structures. Figure 12c shows the SEM images of 0.8 M NaOH-treated samples. The image shows a well arranged micro-fibrillar structure with no agglomeration. Further, increase in molar concentration of the alkali led to increase in the fibril size (see Fig. 12d).

## Conclusion

Chitin has been extracted from periwinkle and snail shells using varying concentrations of acid and alkali.

1. The study shows that CaCO_3_ was completely removed from snail and periwinkle shells when 1.7 M HCl was used for demineralisation.
2. The Degree of deacetylation of the extracted chitin increases as the alkali concentration increases.
3. The study also reveals that the average inter-sheet hydrogen bond energy of the extracted chitin is strongly dependent on acid concentration.
4. It is also clear from the study that the degree of de-acetylation is related to H-Bond energy.
5. The study also shows that extreme high level of the factors (acid and alkaline concentrations) leads to depolymerisation of the polysaccharide chains producing decline in DD and H-Bond.
6. Thermal properties of snail and periwinkle shell chitin are significantly affected by concentration of acid and alkali used in their extraction.

## Electronic supplementary material

Below is the link to the electronic supplementary material.

Supplementary material 1 (DOCX 1060 kb)
