# Supplementary material for: Thermal, structural and acetylation behavior of snail and periwinkle shells chitin
Source: Prog Biomater. 2017 Jul 19;6:97–111. doi: 10.1007/s40204-017-0070-1 (PMC5597570; doi:10.1007/s40204-017-0070-1)
Supplement: Supplementary file 1 — Supplementary material 1 (DOCX 1060 kb) [file 40204_2017_70_MOESM1_ESM.docx]

**Thermal, Structural and Acetylation behaviour of Snail and Periwinkle Shells chitin**

^1^Gbenebor Oluwashina Philips, ^2*^Akpan Emmanuel Isaac and^1^Adeosun Samson Oluropo

^1^Department of Metallurgical and Materials Engineering, University of Lagos, Nigeria

^2^Institut für Verbundwerkstoffe GmbH, 67663, Kaiserslautern, Germany

*Corresponding author. Email: [Emmanuel.Akpan@ivw.uni-kl.de](mailto:Emmanuel.Akpan@ivw.uni-kl.de)

Spectral fittings for treated periwinkle chitin with 1.9 M HCl and (a) 0.4, (b) 0.8 and (c) 1.2 M NaOH

Spectral fittings for treated periwinkle chitin with 1.7 M HCl and (a) 0.4, (b) 0.8 and (c) 1.2 M NaOH

Spectral fittings for snail chitin treated with 1.5 M HCl and (a) 0.4, (b) 0.8 and (c) 1.2 M NaOH

Spectral fittings for snail chitin treated with 1.5 M HCl and (a) 0.4, (b) 0.8 and (c) 1.2 M NaOH

Calculated OH Bonding parameters of Periwinkle Shell Chitin

|  | C(6)–OH…..OH–C(6) | | | NH……...OC | | | C(3)–OH……O–C(5) | | | C(6)–OH…..OC | | |  |
| --- | --- | --- | --- | --- | --- | --- | --- | --- | --- | --- | --- | --- | --- |
|  | Wavenumber | Amount | Bond  Energy | Wavenumber | Amount | Bond  Energy | Wavenumber | Amount | Bond  Energy | Wavenumber | Amount | Bond  Energy | Average  Bond Energy |
| 1. | 3060.67 | 4.43 | 8.92 | 3277.21 | 57.28 | 5.34 | 3432.30 | 29.41 | 2.77 | 3524.83 | 8.88 | 1.24 | 4.57 |
| 2. | 3098.27 | 6.43 | 8.30 | 3252.98 | 28.81 | 5.74 | 3443.77 | 49.96 | 2.58 | 3587.90 | 14.80 | 0.20 | 4.20 |
| 3. | 3097.45 | 3.20 | 8.31 | 3247.15 | 24.39 | 5.83 | 3423.11 | 33.89 | 2.92 | 3523.32 | 38.53 | 1.27 | 4.58 |
| 4. | 3044.79 | 9.07 | 9.18 | 3266.16 | 62.07 | 5.52 | 3429.83 | 22.38 | 2.81 | 3522.72 | 6.48 | 1.28 | 4.70 |
| 5. | 3100.26 | 4.53 | 8.26 | 3253.62 | 27.99 | 5.73 | 3404.11 | 17.55 | 3.24 | 3479.18 | 49.93 | 2.00 | 4.81 |
| 6. | 3107.53 | 4.66 | 8.14 | 3247.32 | 22.34 | 5.83 | 3421.31 | 54.17 | 2.95 | 3500.49 | 18.83 | 1.65 | 4.64 |
| 7. | 3064.80 | 2.19 | 8.85 | 3277.26 | 37.87 | 5.34 | 3403.57 | 11.78 | 3.25 | 3503.46 | 48.17 | 1.60 | 4.76 |
| 8. | 3039.86 | 7.18 | 9.26 | 3245.43 | 59.32 | 5.86 | 3417.23 | 27.82 | 3.02 | 3515.73 | 5.68 | 1.39 | 4.88 |
| 9. | 3081.79 | 2.96 | 8.57 | 3253.86 | 41.58 | 5.72 | 3419.17 | 41.42 | 2.99 | 3527.51 | 14.04 | 1.20 | 4.62 |

Calculated OH bonding of snail shell chitin

|  | C(6)–OH…..OH–C(6) | | | NH……...OC | | | C(3)–OH……O–C(5) | | | C(6)–OH…..OC | | |  |
| --- | --- | --- | --- | --- | --- | --- | --- | --- | --- | --- | --- | --- | --- |
| S/N | Wavenumber | Amount | Bond  Energy | Wavenumber | Amount | Bond  Energy | Wavenumber | Amount | Bond  Energy | Wavenumber | Amount | Bond  Energy | Average  Bond Energy |
| 1. | 3098.76 | 6.61 | 8.29 | 3258.21 | 33.79 | 5.65 | 3440.42 | 42.68 | 2.64 | 3537.77 | 16.92 | 1.03 | 4.40 |
| 2. | 3099.13 | 4.86 | 8.28 | 3252.22 | 19.63 | 5.75 | 3450.10 | 22.59 | 2.48 | 3528.36 | 52.92 | 1.18 | 4.42 |
| 3. | 3098.27 | 6.43 | 8.30 | 3252.94 | 28.80 | 5.74 | 3444.24 | 51.15 | 2.58 | 3593.04 | 13.63 | 0.12 | 4.18 |
| 4. | 3098.92 | 6.30 | 8.29 | 3255.78 | 30.71 | 5.69 | 3458.93 | 61.94 | 2.33 | 3593.73 | 1.05 | 0.10 | 4.10 |
| 5. | 3100.17 | 6.54 | 8.26 | 3258.86 | 34.77 | 5.64 | 3451.69 | 58.21 | 2.45 | 3580.40 | 0.48 | 0.32 | 4.17 |
| 6. | 3100.68 | 5.88 | 8.26 | 3253.93 | 26.64 | 5.72 | 3460.41 | 66.08 | 2.31 | 3596.59 | 1.39 | 0.06 | 4.09 |
| 7. | 3099.41 | 6.68 | 8.28 | 3260.71 | 35.06 | 5.61 | 3456.08 | 57.55 | 2.38 | 3588.14 | 0.72 | 0.20 | 4.12 |
| 8. | 3100.44 | 6.99 | 8.26 | 3260.72 | 33.75 | 5.61 | 3458.20 | 58.23 | 2.34 | 3588.47 | 1.03 | 0.19 | 4.10 |
| 9. | 3100.18 | 6.58 | 8.26 | 3256.55 | 31.86 | 5.68 | 3457.15 | 60.55 | 2.36 | 3589.45 | 1.00 | 0.17 | 4.12 |

TGA of periwinkle chitin treated with 1.7 M HCl and varying alkali concentration

DTG of periwinkle chitin treated with 1.7 M HCl and varying alkali concentration

TGA of periwinkle chitin treated with 1.9 M HCl and varying alkali concentration

DTG of periwinkle chitin treated with 1.9 M HCl and varying alkali concentration

TGA of snail chitin treated with 1.7 M HCl and varying alkali concentration

DTG of snail chitin treated with 1.7 M HCl and varying alkali concentration

TGA of snail chitin treated with 1.9 M HCl and varying alkali concentration

DTG of snail chitin treated with 1.9 M HCl and varying alkali concentration
